# Contextual Detail, but Not Age, Reduces Memory Illusions During Free Recall

**DOI:** 10.1093/geroni/igaf122.3564

**Published:** 2025-12-31

**Authors:** Alana DeLawter, Sara Lute

**Affiliations:** University of North Carolina Asheville, Asheville, North Carolina, United States; University of North Carolina Asheville, Asheville, North Carolina, United States

## Abstract

Research exploring reduced memory illusions is helpful for people of all ages and applies to everyday scenarios. However, this research may be most beneficial for older adults, as they are more likely to show recall memory illusions compared to younger adults (Balota et al., 1999; Pierce et al., 2004). Illusions vary depending on presentation and age, with images accompanying word lists being a benefit (Israel and Schacter, 1997; Smith et al., 2015; Smith and Hunt, 1998). Additionally, increased context could be partially effective in reducing memory illusions for older adults (Skinner & Fernandes, 2010). To test this, we employed the Deese–Roediger–McDermott (DRM) paradigm and measured word list recall across four presentation types (N = 235). Older participants (60-81 years old) and younger participants (18-35 years old) were randomly assigned to one of four groups: an audio-only group, an audio with text group, an audio with low-context image group, or an audio with high-context image group. Results indicated a main effect for presentation type F(3, 227) = 2.82, p = 0.04, η² = 0.035, where participants presented with contextually rich images had fewer memory illusions than participants in the text condition. However contrary to our predictions there was no main effect of age and no interaction between age and presentation type. These findings highlight the importance of information presentation and the encoding process regardless of age.

